# Enhancing H_2_O_2_ Tolerance and Separation Performance through the Modification of the Polyamide Layer of a Thin-Film Composite Nanofiltration Membrane by Using Graphene Oxide

**DOI:** 10.3390/membranes11080592

**Published:** 2021-07-31

**Authors:** Yi-Li Lin, Nai-Yun Zheng, Yu-Shen Chen

**Affiliations:** Department of Safety, Health and Environmental Engineering, National Kaohsiung University of Science and Technology, Kaohsiung 82445, Taiwan; naiyun@nkust.edu.tw (N.-Y.Z.); f108107114@nkust.edu.tw (Y.-S.C.)

**Keywords:** polyamide modification, interfacial polymerization, graphene oxide, hydrogen peroxide resistance, pharmaceutical and personal care product, thin-film composite membrane

## Abstract

Through interfacial polymerization (IP), a polyamide (PA) layer was synthesized on the top of a commercialized polysulfone substrate to form a thin-film composite (TFC) nanofiltration membrane. Graphene oxide (GO) was dosed during the IP process to modify the NF membrane, termed TFC-GO, to enhance oxidant resistance and membrane performance. TFC-GO exhibited increased surface hydrophilicity, water permeability, salt rejection, removal efficiency of pharmaceutical and personal care products (PPCPs), and H_2_O_2_ resistance compared with TFC. When H_2_O_2_ exposure was 0–96,000 ppm-h, the surfaces of the TFC and TFC-GO membranes were damaged, and swelling was observed using scanning electron microscopy. However, the permeate flux of TFC-GO remained stable, with significantly higher NaCl, MgSO_4_, and PPCP rejection with increasing H_2_O_2_ exposure intensity than TFC, which exhibited a 3.5-fold flux increase with an approximate 50% decrease in salt and PPCP rejection. GO incorporated into a PA layer could react with oxidants to mitigate membrane surface damage and increase the negative charge on the membrane surface, resulting in the enhancement of the electrostatic repulsion of negatively charged PPCPs. This hypothesis was confirmed by the significant decrease in PPCP adsorption onto the surface of TFC-GO compared with TFC. Therefore, TFC-GO membranes exhibited superior water permeability, salt rejection, and PPCP rejection and satisfactory resistance to H_2_O_2_, indicating its great potential for practical applications.

## 1. Introduction

Membrane separation processes such as forward osmosis–nanofiltration (FO–NF), reverse osmosis–ultrafiltration (RO–UF), and membrane bioreactor (MBR) processes have been widely employed for removing suspended solids, emerging contaminants such as pharmaceuticals and personal care products (PPCPs) and persistent organic pollutants, microorganisms (e.g., bacteria and biofouling), and ion matter (e.g., monovalent, divalent, and large ions) [1,2,3]. Thin-film-composite (TFC) NF and RO membranes are also commonly used for the desalination of brackish water or seawater. TFC membranes consist of a selective top layer, which is typically polyamide (PA) formed through interfacial polymerization (IP), on a porous substrate [4,5]. However, the PA layer of TFC membranes is vulnerable to oxidative membrane cleaning reagents such as chlorine and hydrogen peroxide (H_2_O_2_), which can damage polymeric structures and deteriorate membrane separation [6,7]. Therefore, the modification of PA layers to enhance both the contaminant removal and physicochemical resistance properties of oxidative cleaning reagents is essential and urgent.

Graphene oxide (GO) consists of hydroxyl, epoxide, diols, ketones, and carboxyl functional groups that can significantly alter van der Waals interactions and increase compatibility with organic polymers [8,9]. GO has been used to modify TFC membranes because it has numerous hydrophilic functional groups that can help water molecules intercalate into its interlayer structures, which act as channels for water diffusion to achieve high permeate flux with excellent chemical stability [10,11]. For example, Alammar et al. prepared polybenzimidazole -GO nanocomposite membranes and reported remarkably enhancement in oil-removal efficiency and maintaining permeance, and resistance to biofouling via a polydopamine (PDA) coating [12]. Muscatello et al. reported that the membrane consisting of layered graphene sheets can improve the antifouling viability, antimicrobial effect, and desalination for separations [13]. In the studies incorporating GO for various membrane modifications, they all have one comment point, which is that adding GO with low concentrations has profound enhancement on membrane separation performance [11,14]. For example, Lin et al. indicated that adding 0.0125–0.0175 wt% of GO to the PA support layer of an FO membrane can reduce internal concentration polarization, enhance water flux, and maintain high rejection of salts and dyes [2,15]. Higher doses of GO usually lead to significant aggregation problems, which compromise membrane performance [16,17]. However, most researchers have only reported the favorable water permeability and salt rejection (i.e., NaCl) of the GO-modified top selective layers of TFC membranes (TFC-GO). Few studies have systematically investigated the effects of TFC-GO membranes on the rejection performance of other valence salts and emerging contaminants (i.e., PPCPs) and chemical resistance to oxidative cleaning agents (i.e., sodium hypochlorite (NaOCl) and H_2_O_2_ [18]) for long-term operation, and such properties are crucial for developing membranes suitable for practical applications.

The separation performance of different salt solutions can vary greatly. Lai et al. embedded GO nanosheets on a PA layer to provide nanochannels with higher membrane surface hydrophilicity, permeate flux, and salt rejection (in an aqueous solution of 0.00087 w/v% GO aqueous solution decreased in the order MgSO_4_ > Na_2_SO_4_ > MgCl_2_ ≫ NaCl), as well as greater antifouling properties in dyes (Rose Bengal > Reactive Black 5 > Methyl Blue) [19]. Song et al. prepared a three-dimensional flower NF membrane with a PA layer modified using MIL-101(Cr) nanoparticles with abundant porous topological structures; the membrane exhibited increasing water flux with considerable variation in monovalent and divalent salt rejection (32–97% under 0.75 MPa, decreasing in the order of MgSO_4_ > Na_2_SO_4_ ≫ MgCl_2_ > NaCl) [20]. Therefore, in terms of TFC membrane separation performance, MgSO_4_ and NaCl as monovalent and divalent salts, respectively, have gained increasing attention in several applications, including desalination, water purification, and wastewater treatment [20,21,22]. Moreover, trace emerging contaminants, namely PPCPs, have been detected in groundwater and seawater and could not be completely removed in traditional treatment processes (i.e., drinking water/wastewater) [23,24]. Therefore, dense RO and NF membranes have attracted considerable attention for their capability to mitigate biological and organic fouling and improve PPCP removal for water reclamation. However, commonly used chemical cleaning agents, namely NaOCl and H_2_O_2_, can oxidize foulants with complex functional groups such as ketonic, aldehyde, or carboxyl groups [25]. Ling et al. reported that free chlorine species (HOCl and OCl^−^) can damage the PA layer of TFC membranes [26]. Compared with NaOCl, H_2_O_2_ may be preferable because it does not yield toxic by-products after reacting with organics, whereas NaOCl has been proven to form numerous disinfection by-products such as carcinogenic trihalomethanes and absorbable organic halogen [27,28].

In the current study, during PA layer synthesis, GO nanoparticles were embedded through IP on the top of a commercial PSf substrate to form TFC NF membranes by using the optimal dosage determined in our previous study [2]. The objectives of the current study are to enhance the separation performance and oxidant resistance of TFC membranes. The permeate flux, monovalent, and divalent salt rejection (NaCl and MgSO_4_), and PPCP rejection of TFC and TFC-GO membranes were compared before and after intensive H_2_O_2_ exposure. Membrane characteristics were evaluated, including surface morphology and roughness, functional groups, and hydrophilicity before and after intensive H_2_O_2_ exposure. Finally, the mechanisms of GO embedded in PA in the enhancement of PPCP rejection and membrane resistance to H_2_O_2_ were evaluated.

## 2. Materials and Methods

### 2.1. Chemicals and Reagents

**Figure 1:**
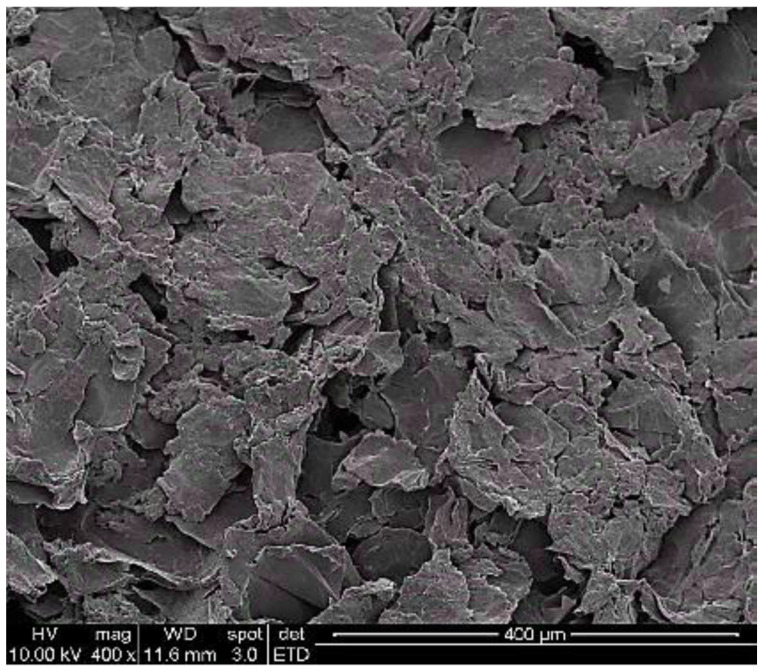
SEM micrograph of GO (400×).

M-phenylenediamine (MPD, 99%, Acros Organics, New Jersey, USA), sodium laurylsulfate (SDS, 90%, Showa Chemical Industry, Tokyo, Japan), trimesoyl chloride (TMC, 99%, Tokyo Chemical Industry, Tokyo, Japan), and hexyl hydride (n-hexane, 99%, Seedchem, Camberwell, Australia) were used for the IP of the PA selective layer. GO was prepared according to the modified Hummers method through the chemical exfoliation of graphite [11] and had flaky and oval shape observed using a scanning electron microscopy (SEM, in Figure 1). The GO sheets were ground into powders and sieved through a 0.45 µm stainless filter before use. Sulfuric acid (H_2_SO_4_, 97%, Honeywell, Charlotte, NC, USA), sodium nitrate (NaNO_3_, 99%, Sigma-Aldrich, St. Louis, USA), potassium permanganate (KMnO_4_, 99%, Showa, Tokyo, Japan), hydrogen peroxide (H_2_O_2_, 30%, Showa, Tokyo, Japan), hydrochloric acid (HCl, 97%, Aencore Chemical Pty., Ltd., Surry Hills, Australia), and ethanol (C_2_H_6_O, 95%, Echo Chemicals Co., Ltd., Miaoli County, Taiwan) were also used in this study.

Sodium chloride (NaCl, 99%, Taiyen, Tainan, Taiwan) and magnesium sulfate (MgSO_4_, 99%, Sigma-Aldrich, St. Louis, USA) were used to evaluate the salt rejection properties of the TFC and TFC-GO membranes. Six PPCPs frequently detected in aqueous environments in Taiwan were selected to evaluate the removal efficiency of the fabricated membranes, including sulfamethazone (SMZ), ibuprofen (IBU), triclosan (TRI), sulfadiazine (DIA), sulfamethoxazole (SMX), and carbamazepine (CBZ). High-purity (>99%) SMZ, IBU, and TRI were purchased from Alfa Aesar (Ward Hill, USA), and DIA, SMX, and CBZ were purchased from MP Biomedicals (Irvine, USA). The physicochemical properties of the selected PPCPs are presented in Table S1.

### 2.2. Preparation of TFC and TFC-GO Membranes

A commercial UF PSf membrane (PSf UF1812; A-spring Technology, Taiwan) was used as a substrate to form the TFC membrane. The physicochemical properties of this membrane are presented in Table 1, which was provided by the manufacturer. The membrane was washed using Milli-Q water and stored at 4 °C in the dark prior to use. PSf is a stable UF membrane for eliminating small-sized particles (<0.02 μm). The PA top layer was intensively polymerized on the PSf substrate using 2.0 wt% MPD solution with 0.1 wt% SDS in deionized (DI) water and 0.1 wt% TMC solution (in n-hexane) [2]. First, the MPD solution was poured on the PSf substrate and allowed to react for 3 min, after which the excess MPD solution was removed with a rubber scraper. Next, the TMC solution was gently poured on top to react with the residual MPD for 3 min to form the PA active layer and complete TFC membrane fabrication. The TFC-GO membrane was prepared with 0.015 wt% GO in the MPD solution for incorporating GO nanoparticles into the PA layer. We did not explore the effect of GO concentration on the oxidant resistance of the membrane in this study but adopted the optimal GO dosage developed in our previous study [29]. The TFC and TFC-GO membranes were thoroughly rinsed using DI water to remove residual monomers and were air-dried for 5 min, after which the surface of the membrane was activated using an oven at 80 °C for 10 min. Finally, the prepared TFC-PA membrane was stored in DI water until further experiments were conducted.

### 2.3. Filtration Experiments

The performance of the TFC and TFC-GO membranes was tested using three laboratory-scale parallel rectangular cross-flow filtration modules composed of 316 stainless steel [23]. Membrane coupons with a surface area of 137.75 cm^2^ were pre-compacted for 6 h using DI water to reach a steady-state permeate flux (L/h-m^2^) before the start of each experiment. The filtration system was operated in the recycling mode with 30 L of feed solution supplied using a high-pressure pump (Hydracell, Wanner Engineering Inc., USA). The experiment was performed under a constant cross-flow velocity of 0.1 m/s, a transmembrane pressure of 100 psi, and a temperature of 25 ± 0.5 °C. The schematic and detailed specifications are presented in Figure S1 and Table S2. In our preliminary experiments for a longer period of time (to 3 days), membrane performance reached stability within 1 day and was maintained in the following 2 days. Therefore, we shortened the separation experiments to 24 h, which meets a commonly adopted experimental period in the literature [3,30,31]. To evaluate the salt rejection performance, experiments were conducted using the feed solution comprising 1 g/L NaCl or MgSO_4_ at the same aforementioned cross-flow velocity, transmembrane pressure, and temperature. The schematic variation of permeate flux with time during filtration is displayed in Figure S2.

According to the acidic dissociation constant (p*K*_a_) and hydrophobicity (log*_Kow_*) at pH 7, the target PPCPs were classified as ionic (I) or nonionizable (N) and hydrophobic (HPO) or hydrophilic (HPI) PPCPs. The PPCP rejection properties of the TFC and TFC-GO membranes were assessed using 30 L of feed solution containing 800 μg/L of each PPCP and background electrolytes (20 mM NaCl and 1 mM NaHCO_3_), and the results after operating for 24 h were reported. The rejection of salts and PPCPs was calculated as (1 − C_p_/C_f_) × 100%, where C_p_ and C_f_ are the concentrations of each target compound in the permeate and feed solutions, respectively.

### 2.4. Membrane Resistance to H_2_O_2_ Oxidation

To assess the antioxidant properties of the TFC and TFC-GO membranes, the PA active layer of each membrane was exposed to 4000 ppm H_2_O_2_ solution for 0, 1, 4, 7, and 24 h to simulate field operations under a low H_2_O_2_ concentration with a long period of contact times [32,33]. In addition, total H_2_O_2_ exposure is expressed as ppm-h, namely 0, 4000, 16,000, 28,000, and 96,000 ppm-h, which were calculated for H_2_O_2_ concentrations by multiplying the exposure times. The pH of the H_2_O_2_ solution was adjusted to 7 with 0.1 N HCl. After the designated period, the membrane was taken out and washed with DI water to remove residual H_2_O_2_ on the surface. Next, the H_2_O_2_-exposed TFC and TFC-GO membranes were evaluated for permeate flux, salt rejection, and PPCP rejection through the filtration experiment described in Section 2.3.

### 2.5. Analytical Methods

The TFC and TFC-GO membranes were completely dried before their analyses. The surface area of functional groups was measured using attenuated total reflectance Fourier transform infrared spectroscopy (ATR-FTIR, Spectrum 100, PerkinElmer, UK), and hydrophilicity was measured using a contact angle meter (Phx mini, Phoenix, Suwon City, Korea). The contact angle and thickness of each membrane were reported as the average values of at least five measurements applied at random sites. The ATR-FTIR spectrum was averaged using 40 scans with a resolution of 4 cm^−1^ and a scanning range of 400–4000 cm^−1^ in the absorbance mode. The ratio of the intensities of the bands at 1547 and 1587 cm^−1^ (I_1547_/I_1587_) in the ATR-FTIR spectra were used to confirmed the degree of polymerization of the PA layer. The bands at 1547 and 1587 cm^−1^ indicated the stretching vibration of amide II (N–H and C–N) in the ATR-FTIR spectra and aromatic ring stretching in the supporting PSf, respectively [10,34]. Membrane samples were sputtered with a thin layer of gold (Au) for surface morphology and cross-section analysis using scanning electron microscopy (SEM, ESEM Quanta 200, Graz, USA). Regarding the roughness variation of the membranes, we have used the Image J software (Version 1.53k) to analyze membrane surface roughness using the SEM images.

The salt concentrations of NaCl and MgSO_4_ were measured using a conductivity meter (SC-110, Suntex, Kaohsiung, Taiwan). PPCP concentrations were analyzed using high-performance liquid chromatography (LC-20A Prominence, Shimadzu, Kyoto, Japan) according to our previous method [23,35]. The pH was measured using a frequently calibrated pH meter (FE20-FiveEasy, Mettler Toledo, Greifensee, Switzerland). To evaluate PPCP adsorption, a methanol extraction method was used, which is described in detail in our previous study on mechanically separated PA + PSf and polyethylene terephthalate (PET) layers [36].

## 3. Results and Discussion

### 3.1. Effects of Modification on the Physicochemical Characteristics of TFC Membrane

#### 3.1.1. Surface Morphology

**Figure 2:**
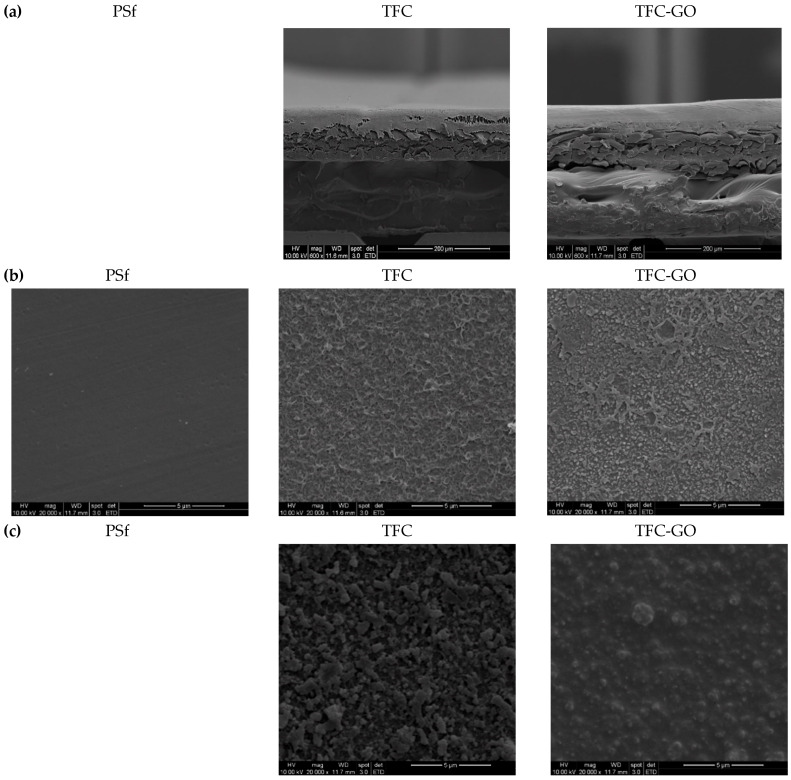

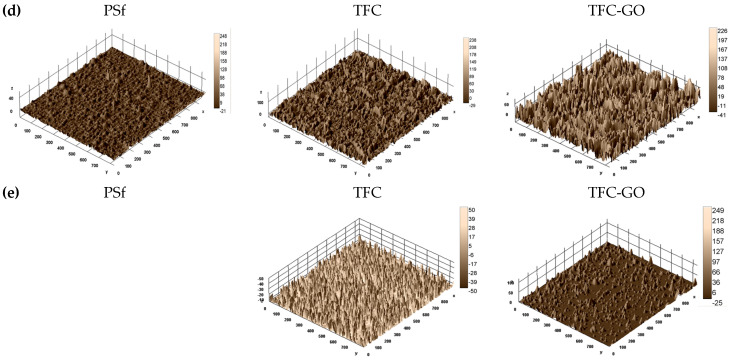
SEM pictures of PSf, TFC, and TFC-GO membranes: (**a**) cross-section, (**b**) membrane surface before and (**c**) after H_2_O_2_ exposure (20,000×), and (**d**) 3D images before and (**e**) after H_2_O_2_ exposure.

The cross-section morphology and top surface of all membranes were characterized using SEM, and the related images are displayed in Figure 2a–c. The 3D images of TFC and TFC-GO membranes before and after H_2_O_2_ exposure, respectively, are displayed in Figure 2d,e. The thickness of the TFC-GO cross-section slightly increased compared with that of the TFC (Figure 2a), indicating that GO molecules interacted with MPD and TMC molecules to enhance the crosslinking structures of the PA layer, resulting in increased thickness and compactness of the PA active layer [37]. The morphology of the membrane has been suggested to be strongly dependent on the thickness of the PA layer [38]. The PSf membrane had a highly smooth surface (Figure 2b,d), whereas the TFC and TFC-GO membranes exhibited ridge-valley structures with some protuberances (Figure 2d), which comprise the characteristic morphology of PA layers [39] and imply the successful formation of the top layer. In particular, the TFC-GO membrane presented significantly broadened ridge–valley structures and a slight agglomeration of GO nanoparticles on the membrane surface when compared with the TFC membrane (Figure 2b). The SEM images of the surface morphology of the TFC and TFC-GO membranes after intensive exposure to H_2_O_2_ are presented in Figure 2c. For the pristine TFC membrane (Figure 2c,e), significant swelling and deformation of the PA layer occurred because the strong oxidant broke down its polymer structure. However, the surface of the TFC-GO membrane (Figure 2c,e) exhibited moderate swelling, which confirmed the robust protection efficiency and superior stability of GO incorporated into the PA layer [14]. The TFC-GO membrane after H_2_O_2_ exposure had a slightly decreased Rq and Ra (5.8 μm and 4.1 μm; Table 2), which confirmed a significant decline in roughness. These results suggest that H_2_O_2_ exposure might lead to OH·radical formation on the membrane surface, the degradation of which is accelerated by swelling of the PA layer, causing the uncoated TFC membrane to be more vulnerable to the oxidant [26]. However, when the PA RO membrane was exposed to 248 mg/L chlorine for 10 h, the tensile strength of the PA layer decreased, resulting in the formation of a breached area on the membrane surface [6].

#### 3.1.2. Surface Functional Groups

**Figure 3:**
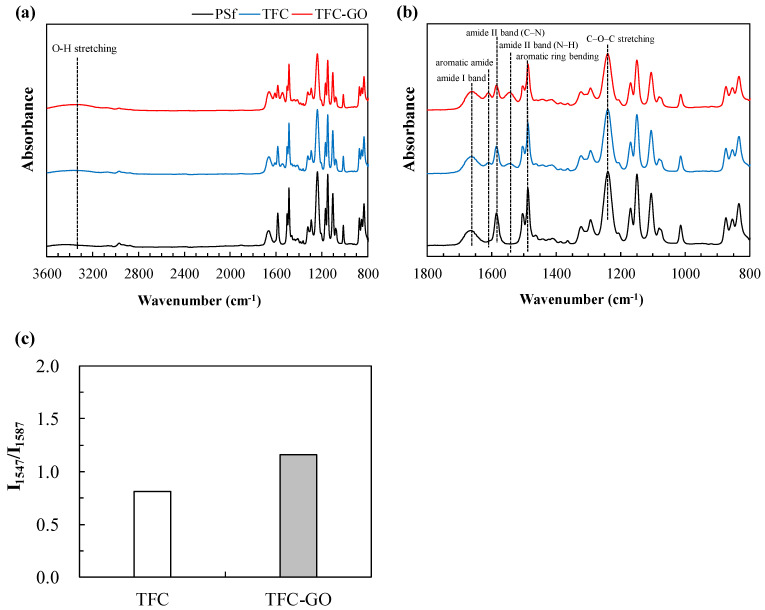
ATR-FTIR spectra of PSf, TFC, and TFC-GO membranes: (**a**) full scan with the wavenumbers in the range of 800–3600 cm^−1^, (**b**) zoom in with the wavenumbers in the range of 800–1800 cm^−1^, and (**c**) degree of grafting TFC and TFC-GO membranes.

**Figure 4:**
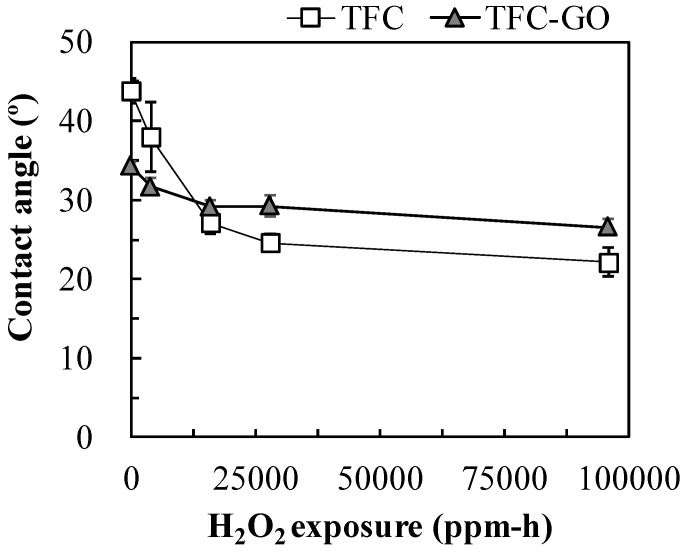
Contact angle of TFC and TFC-GO membranes with the range of 0−28,000 ppm-h H_2_O_2_ exposure. Error bars represent one standard deviation of triplicate measurements.

**Figure 5:**
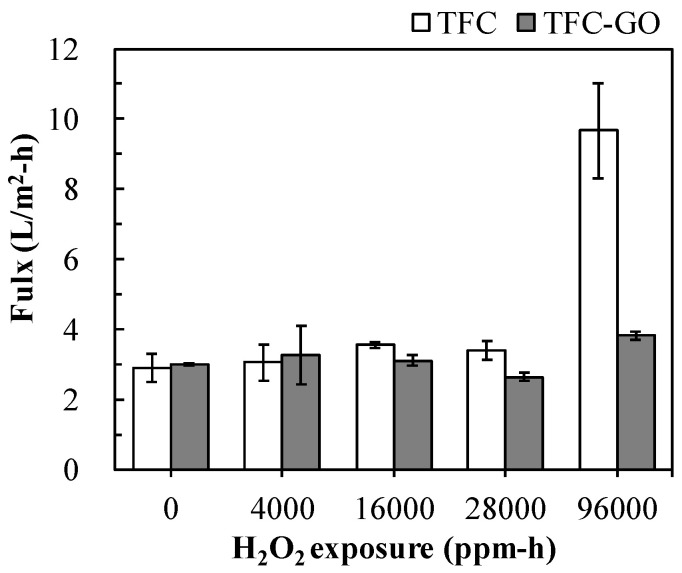
Permeate flux of TFC and TFC-GO membranes with the range of 0−28,000 ppm-h H_2_O_2_ exposure. Error bars represent one standard deviation of triplicate measurements.

The full scan and zoomed images from the ATR-FTIR of the PSf, TFC, and TFC-GO membranes are presented in Figure 3a,b, and the degree of polymerization is presented as the ratio of intensities at 1547 and 1585 cm^−1^ (I_1547_/I_1585_) in Figure 3c. Compared with those of the PSf membrane, the spectra of the TFC and TFC-GO membranes had weaker intensities, and several peaks disappeared because of the formation of extra chemical groups on the membrane surface following PA polymerization (Figure 3a), which is consistent with the results of previous studies [4,37]. The primary amide I band (C=O stretching) at 1664 cm^−1^, aromatic amide (N–H) at 1609 cm^−1^, amide I band (C=O stretching vibration) at 1659 cm^−1^, and amide II band (in-plane N–H bending and C–N stretching vibrations) at 1547 cm^−1^ (Figure 3b) were observed [4,10,34], indicating successful PA layer formation. The characteristic peak included amide band breathing at 795 cm^−1^ and carboxylic acid (C=O stretching and O–H bending) at 1450 cm^−1^, which are associated with PA polymerization caused by amide functional groups [4,10,34]. Moreover, the spectra exhibited C–O–C asymmetric stretching at 1245 cm^−1^ and aromatic in-plane ring bending stretching at 1488 cm^−1^, indicating gradual weakening following the IP of the PA layer and the successful polymerization of PA in the PSf layer. The peaks at 3300 cm^−1^ represent the stretching vibrations of the O–H groups attached to the basal plane of the GO structure [10,11,14]. Although the intensity of this peak was weak because of the small amount of GO, it may provide evidence of the successful incorporation of GO nanoparticles into the PA layer (Figure 3c, Figure 4, and Figure 5). Ling.et al. exposed their TFC membranes to 2–50 mM H_2_O_2_ for 16–24 days (which equals to 26,112–979,200 ppm-h H_2_O_2_ exposure intensity) and did not observe significant changes of the polyamide functional groups of the amide I band and aromatic amide band II [26]. Considering the H_2_O_2_ exposure intensity was 4000–100,000 ppm-h in our study, we do not expect significant changes of ATR-FTIR results of the membranes before and after H_2_O_2_ exposure.

Figure 3c presents the degree of polymerization (I_1547_/I_1585_) used to quantify the degree of grafting, which increased from 0 for the PSf to 0.6 for the TFC and 1.0 for the TFC-GO. These features confirm the effective crosslinking of MPD and TMC in the TFC membrane, and the effective incorporation of GO nanoparticles enhanced the grafting of PA structures in the TFC-GO membrane.

#### 3.1.3. Surface Hydrophilicity

Figure 4 displays the contact angle of the TFC and TFC-GO membranes after an H_2_O_2_ exposure intensity of 0–96,000 ppm-h. In the absence of H_2_O_2_ exposure, the contact angle of the TFC-GO membrane (34.2°) was much lower than that of the TFC membrane (43.8°), indicating enhanced surface hydrophilicity. The contact angle of the prepared membranes in this study was superior than those of Chae et al. embedding 0, 15, 38, and 76 ppm GO in the PA layer of a TFC membrane with contact angle of 47.0°–75.0° [11]. With increasing H_2_O_2_ exposure intensity, the contact angle of the TFC membrane significantly decreased to 37.9° and gradually decreased to 22.1° at H_2_O_2_ exposure intensities of 4000 and 96,000 ppm-h, respectively. However, the contact angle of the TFC-GO membrane only slightly decreased to 26.5° and 31.7° after H_2_O_2_ exposure intensities of 4000 and 96,000 ppm-h, respectively. The results correlated well to low surface swelling, deformation of the TFC-GO observed using SEM (Figure 3c), and the increase permeate flux (Figure 5). Furthermore, the contact angle of both membranes after H_2_O_2_ exposure was much lower than those of the unexposed membranes, which is consistent with the findings of a study involving PA RO membranes exposed to chlorine [6]. The observed hydrophilicity increase could be attributed to the unbalanced dipole moments induced at the surface chains caused by surface oxidation [6,25]. H_2_O_2_ can form OH radicals on the membrane surface, which can cause the PA structure to be more vulnerable and can accelerate its degradation, thereby causing swelling and deformation of the PA layer [26]. The contact angle of the TFC-GO membrane was more stable than that of the TFC membrane, which may be due to the π–π interactions between graphitic domains, which can mitigate damage to the PA layer [40]. Chae et al. indicated that the TFC membranes embedding GO in the PA layer exhibited smoother surface with increasing hydrophilicity and negative surface charge [11]. These results demonstrated that incorporating GO nanoparticles into the PA layer modified the membrane surface characteristic and successfully enhanced its tolerance to H_2_O_2_, thereby increasing its chemical stability and surface hydrophilicity.

### 3.2. Effects of Modification on Permeate Flux and Salt Rejection

Figure 5 presents the permeate flux of the TFC and TFC-GO membranes after H_2_O_2_ exposure intensities of 0−96,000 ppm-h. Although the TFC-GO membrane exhibited slightly increased membrane thickness, the presence of GO provided extra channels in the dense PA layer for water molecules to pass through, resulting in increased permeate flux compared with that of the TFC membrane [10,11]. The other reason could be the restricted compaction of membrane polymer matrix due to the presence of GO [41]. With increasing H_2_O_2_ exposure intensity, the permeate flux of the TFC membrane significantly increased from 3.1 to 9.7 L/m^2^-h, with high variation in membrane performance (large error bar), especially after the 96,000 ppm-h H_2_O_2_ exposure intensity. The phenomenon could be due to PA structure damage caused by H_2_O_2_ (Figure 2c) and the decreasing contact angle of the TFC membrane, as shown in Figure 4. In addition, the stable permeate flux of the TFC-GO membrane (2.6–3.8 L/m^2^-h) indicates its superior chemical stability under intensive H_2_O_2_ exposure. Furthermore, GO has abundant negatively charged groups, which act as an acceptor of OH radicals, resulting in a decrease in the number of oxidant radicals exposed to the PA layer, thereby increasing the H_2_O_2_ resistance of the membrane [18,42]. Thus, the TFC-GO membrane demonstrated highly stable permeate flux and mitigated damage to the membrane structure caused by H_2_O_2_, thereby extending its lifetime. Ling.et al. exposed their TFC membranes to 2–50 mM H_2_O_2_ for up to 24 days and found that the permeate flux was stable in 50 mM H_2_O_2_ exposure for at least 18 days of operation, corresponding to >734,400 ppm-h H_2_O_2_ tolerance [26]. On the other hand, Fei et al. covalently anchored GO to the hydroxyl groups of the PBI membranes for molecular separations in organic solvents and reported considerably improved solvent flux and long-term stability under continuous operation of over 14 days [41]. It can be speculated from the above-mentioned results that the prepared TFC-GO membrane could be robust and maintain a long-term stable performance with continuous operation in a cross-flow filiation.

**Figure 6:**
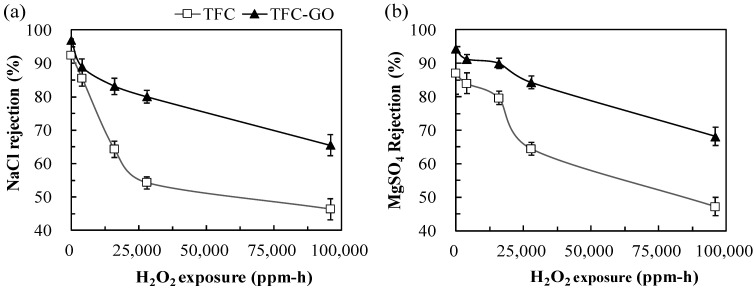
(**a**) NaCl and (**b**) MgSO_4_ rejection by TFC and TFC-GO membranes with the range of 0−28,000 ppm-h H_2_O_2_ exposure. Error bars represent one standard deviation of triplicate measurements.

Figure 6 presents the NaCl and MgSO_4_ rejection of TFC and TFC-GO after H_2_O_2_ exposure intensities of 0−96,000 ppm-h. Both membranes exhibited a higher rejection rate for the divalent salt (MgSO_4_; Figure 6b) compared with the monovalent salt (NaCl; Figure 6a), which can be explained by the Gibbs–Donnan effect occurring between the solutes and the membrane surface charge [19]. According to the Gibbs–Donnan effect principle, the salt rejection properties of containing high-valent anions (e.g., Na_2_SO_4_ and MgSO_4_) are greater than those of containing low-valent anions (e.g., MgCl_2_ and NaCl) for negatively charged polyamide NF–RO membranes [19,40]. Thus, the Gibbs–Donnan effect was indicated by the TFC-GO membrane exhibiting a higher NaCl and MgSO_4_ rejection rate (94.3% and 96.9%, respectively) in the absence of H_2_O_2_ when compared with the TFC membrane (87.0% and 92.3%, respectively). As H_2_O_2_ exposure intensities were increased, the salt rejection properties of both membranes decreased, especially in the TFC membrane. Moreover, the TFC-GO membrane maintained a considerably higher rejection rate for both salts after the 96,000 ppm-h H_2_O_2_ exposure intensity than the TFC membrane did. Shao et al. indicated that the embedment of GO in the PA layer of membrane could increase ion transmission resistance, which further lad to the decrease of Na^+^ concentration in the permeate side, thus increasing salt rejection [42]. Therefore, incorporating GO into PA layers with increasing oxidant resistance enhanced salt rejection. Although the permeate flux of the TFC-GO membrane after H_2_O_2_ exposure (2.6–3.8 L/m^2^-h) in this study was lower than that of Shao et al., who used the spin-coating method to coat GO on a TFC-RO membrane (50–150 L/m^2^-h) with 6000 mg/L chlorine exposure for 16 h, the NaCl rejection of the TFC-GO membrane (96.9–65.5%) with increasing H_2_O_2_ exposure intensity in this study was superior to that of the above-mentioned literature (95.3–63.0%) [42]. Overall, the TFC-GO membrane exhibited increases in hydrophilicity (Figure 4), NaCl and MgSO_4_ rejection (Figure 6), and stable permeate flux (Figure 5), which would be advantageous in numerous practical water treatment applications. No trade-off between rejection and permeation was observed in this study, which was also reported in the study of Zhang et al. using graphene oxide/carbon nanotube membranes [43].

### 3.3. Effects of Modification on Permeate PPCP Rejection

**Figure 7:**
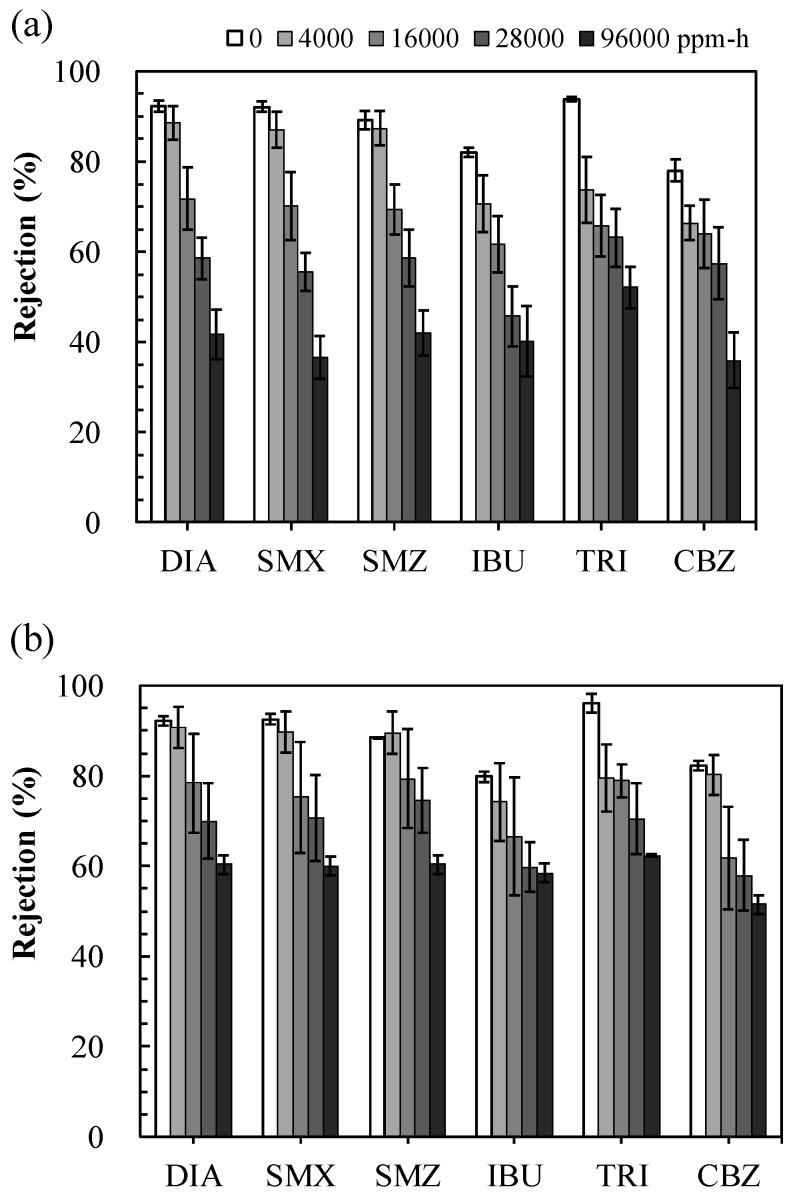
The removal of PPCP by (**a**) TFC and (**b**) TFC-GO membranes with the range of 0−28,000 ppm-h H_2_O_2_ exposure. Error bars represent one standard deviation of triplicate measurements. SMX: sulfamethoxazole, DIA: sulfadiazine, IBU: ibuprofen, SMZ: sulfamethazine, TRI: triclosan, CBZ: carbamazepine.

Figure 7 presents the removal of PPCPs by the TFC and TFC-GO membranes under H_2_O_2_ exposure intensities of 0−96,000 ppm-h. The dissociated and highly hydrophilic compounds DIA (p*K*_a_ = 6.4 and log *K*_ow_ = 0.21), SMX (p*K*_a_ = 5.7 and log *K*_ow_ = 0.86), and SMZ (p*K*_a_ = 7.6 and log *K*_ow_ = 1.62) were effectively removed by both the TFC and TFC-GO membranes because of electrostatic repulsion and the steric hindrance (sieving) effect demonstrated in our previous studies [3,35]. The hydrophobic and ionized IBU (p*K*_a_ = 4.3 and log *K*_ow_ = 3.14), with a molecular weight of only 151 Da, was also moderately removed through electrostatic repulsion [3]. The highly hydrophobic nonionized TRI (p*K*_a_ =8.0 and log *K*_ow_ = 4.86) and CBZ (p*K*_a_ = 13.9 and log *K*_ow_ = 2.45) were moderately removed through steric hindrance along with membrane adsorption and penetration through membrane pores [23,44], which is discussed in Section 3.4. With increasing H_2_O_2_ exposure intensity, both membranes exhibited decreased removal for all PPCPs, which was similar to that for salt rejection (Figure 6), especially the pristine TFC membrane. PPCP rejection of the TFC-GO membrane was 10–24% higher than that of the TFC membrane when H_2_O_2_ exposure intensity was increased from 0 to 96,000 ppm-h. Moreover, the PPCP rejection properties of the TFC-GO membrane (52–60%) remained stable compared with the TFC membrane (36–52%) under the high-intensity H_2_O_2_ exposure of 96,000 ppm-h. This phenomenon demonstrated that GO can capture active radicals and H_2_O_2_, resulting in the alleviation of damage to membrane structures [14,18]. In addition, GO nanoparticles can protect the underlying PA from oxidation exposure [10] and enhance the mechanical stability of membranes [45]. Therefore, the GO embedded in the active layer was efficient in enhancing the oxidant resistance and PPCP rejection properties of the TFC-GO membrane. Therefore, the TFC-GO membrane demonstrated remarkable resistance to high-intensity H_2_O_2_ exposure with increases in PPCP rejection through synergistic contributions.

### 3.4. Effects of Modification of PPCP Adsorption on the Membrane Surface

**Figure 8:**
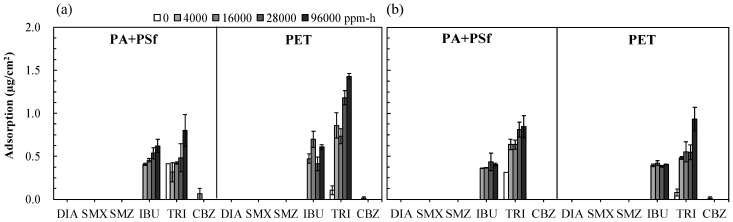
Adsorption of IBU, TRI, and CBZ extracted from PA + PSf and PET layers of (**a**) TFC and (**b**) TFC-GO membranes with the range of 0−28,000 ppm-h H_2_O_2_ exposure. Error bars represent one standard deviation of triplicate measurements.

PPCP reportedly adsorbs onto the membrane surface or the fouling layer, which may cause the cake-enhanced concentration polarization phenomenon [23]. After the PPCP rejection experiments, each membrane was mechanically separated from the PA layer by using PSf and the nonwoven PET layer to extract PPCPs from different membrane layers and verify whether the mechanisms of adsorption and penetration occurred. Figure 8 displays the adsorption of PPCPs onto different TFC and TFC-GO layers under an H_2_O_2_ exposure intensity of 0−96,000 ppm-h. Three crucial findings were obtained. First, only the highly hydrophobic TRI and IBU were adsorbed onto both the TFC and TFC-GO membranes in both the PA + PSf and PET layers. Moreover, only trace amounts of the hydrophobic nonionized CBZ could be extracted from both membranes under the high-intensity H_2_O_2_ exposure of 4000 ppm-h (i.e., an H_2_O_2_ exposure time of 1 h). The membrane surface with an organic hydrophobic polymer PA was demonstrated to adsorb the hydrophobic PPCP through surface affinity [46]. The adsorption amounts on the PA + PSf and PET layers for both membranes (0.5 ± 0.2 µg/cm^2^) were significantly lower than those of the commercialized TFC NF and RO membranes (i.e., NF270, NF90, and XLE) in the range of 20–36 and 2 ± 0.1 µg/cm^2^ for PA + PSf and PET layers [3,35], which may have been due to additional modification of the functional groups on the PA layer. Second, the adsorption of TRI and IBU gradually increased as H_2_O_2_ exposure intensity increased for both the TFC and TFC-GO membranes. These results may be due to the hydrogen bond and electrostatic interaction between the high adsorption of TRI and IBU onto the membrane surface, which cannot prevent the significant penetration of PPCPs through the membranes after extended exposure to H_2_O_2_, which results in attachment or adsorption onto the membranes’ surfaces [47]. Third, the adsorption of TRI and IBU onto the TFC-GO membrane was slightly lower than that of those extracted from the TFC membrane. These results suggest that GO incorporation into the PA layer increased the negative charge of the membrane surface, resulting in an increase in the electrostatic repulsion of the negatively charged PPCPs [5].

## 4. Conclusions

A PA layer was synthesized using the IP method on the top of a commercialized PSf substrate to form a TFC nanofiltration membrane, which was coated with GO (TFC-GO) to enhance oxidant resistance and membrane performance. TFC-GO exhibited increased surface hydrophilicity, water permeability, salt rejection, PPCP removal efficiency, and H_2_O_2_ resistance when compared with TFC. Under H_2_O_2_ exposure, the surfaces of the TFC and TFC-GO membranes were damaged and swollen. Notably, the permeate flux of the TFC-GO remained stable with significantly higher NaCl, MgSO_4_, and PPCP rejection under H_2_O_2_ exposure intensities than did TFC, which exhibited a 3.5-fold flux increase with an approximate 50% decrease in salt and PPCP rejection. These results suggest that GO incorporated into the PA layer could react with oxidants to mitigate membrane surface damage and increase the negative charge on the membrane surface, resulting in the enhanced electrostatic repulsion of negatively charged PPCPs. This hypothesis was confirmed by the significant decrease in PPCP adsorptions on the surface of TFC-GO compared with TFC. TFC-GO membranes exhibited superior water permeability, salt rejection, PPCP rejection, and satisfactory resistance to H_2_O_2_, indicating great potential for practical applications.

## Figures and Tables

**Table 1:** Physicochemical characteristics of the PSf substrate.

Property	UF
Manufacture	A-spring Technology
Membrane type	UF
Membrane material	Polysulfone
Pure water permeability (L/m^2^ h bar)	379.2
Contact angle (°)	60.4 ± 3.3
Average pore size (nm)	20
The pH range of operation	2–13
NaCl rejection (%)	1.4
MgSO_4_ rejection (%)	2.2

**Table 2:** Surface characteristics of the fabricated TFC and TFC-GO membranes.

Membrane	Surface Morphology	Rq ^a^ (μm)	Ra ^b^ (μm)
TFC before H_2_O_2_	Ridge–valley	11.2	8.9
TFC-GO before H_2_O_2_	Ridge–valley	17.9	14.5
TFC after H_2_O_2_	Ridge–valley	11.8	9.8
TFC-GO after H_2_O_2_	Smooth valley	5.8	4.1

^a^ Root mean square deviation of surface roughness. ^b^ Arithmetical mean deviation of surface roughness.

## Data Availability

Not applicable.

## Supplementary Materials

The following are available online at https://www.mdpi.com/article/10.3390/membranes11080592/s1, Table S1: Physicochemical properties of the selected PPCPs in this study, Table S2: The parameters and the detailed specifications for the parallel rectangular cross-flow filtration system, Figure S1: The schematic diagram of the cross-flow filtration system. Figure S2: The schematic variation of permeate flux with filtration time.
