# Supplementary material for: Enhancing H2O2 Tolerance and Separation Performance through the Modification of the Polyamide Layer of a Thin-Film Composite Nanofiltration Membrane by Using Graphene Oxide
Source: Membranes (Basel). 2021 Jul 31;11(8):592. doi: 10.3390/membranes11080592 (PMC8398487; doi:10.3390/membranes11080592)
Supplement: Supplementary file 1 [file membranes-11-00592-s001.zip › membranes-1293474-supplementary.pdf]

Supplementary Information

Enhancing H_2O_2 tolerance and separation performance through the modification of the polyamide layer of a thin-film composite nanofiltration membrane by using graphene

Yi-Li Lin[a]*, Nai-Yun Zheng[a], Yu-Shen Chen[a]

[a]*Department of Safety, Health and Environmental Engineering, National Kaohsiung University of Science and Technology, Kaohsiung 82445, Taiwan*

*Corresponding author: Yi-Li Lin

E-mail: yililin@nkust.edu.tw

Phone: +886-7-6011000#32328

Fax: +886-7-6011061

Address: No. 1, University Rd., Yanchao Dist., Kaohsiung City 824, Taiwan.

This document consists of 4 pages including 2 Tables and 1 Figure.

Table S1 Physicochemical properties of the selected PPCPs in this study.

Name (Abbreviations)	Structure	Molecular formula	Molecular weight (g/mol)	Diffusion coefficient (10^{-10} m^2/s)[a]	Stokes radius (nm) [a]	p*K*a[b]	logK_{ow}[c]	Classif-ication[d]
Carbamazepine (CBZ)	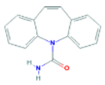	$C_{15}H_{12}N_2O$	236.3	5.81	0.422	13.9	2.45	HPO-N
Ibuprofen (IBU)	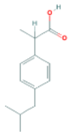	$C_{13}H_{18}O_2$	206.3	5.95	0.412	4.3	3.14	HPO-I
Sulfadiazine (DIA)	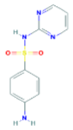	$C_{10}H_{10}N_4O_2S$	250.3	6.11	0.401	6.4	0.21	HPI-I
Sulfamethoxazole (SMX)	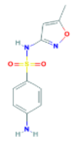	$C_{10}H_{11}N_3O_3S$	253.3	6.08	0.403	5.7	0.86	HPI-I
Sulfamethazine (SMZ)	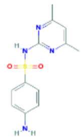	$C_{12}H_{14}N_4O_2S$	278.3	5.58	0.439	7.6	1.62	HPI-N
Triclosan (TRI)	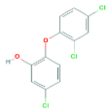	$C_{12}H_7Cl_3O_2$	289.5	5.91	0.415	8.0	4.86	HPO-N

[a] Calculated from the method proposed by Wilke and Chang [1].
[b] ADME/Tox Web Software.
[c] Calculated using ChemOffice 2010.
[d] HPI: hydrophilic (log K_{ow} ≦ 2), HPO: hydrophobic (log K_{ow} > 2), I: ionic (pK_a ≦ 7), N: non-ionic (pK_a > 7).

Table S2 The parameters and the detailed specifications for the parallel rectangular cross-flow filtration system are given in the following:

No	Apparatus or instrument	Specification
1	Water bath	Model：Water Bath D-606, DENG YNG, Taiwan.<br>Control the feed water temperature at 25 ± 0.5 ℃.
2	Feedwater reservoir	30 L, polyethylene (PE), Taiwan.
	Cross-flow filtration module	A self-designed, cross-flow mode filtration apparatus with a flat-sheet membrane cell.<br>All parts of the experimental apparatus were made of stainless steel.<br>The effective membrane area：137.75 cm^2.
3	Pressure gauge	Operating condition：6.9 kg/cm^2 (100 psi).
4	Flow meter	Operating condition: 1.14 L/min.
5	Pressure regulation valve	1/4” Stainless steel 316.

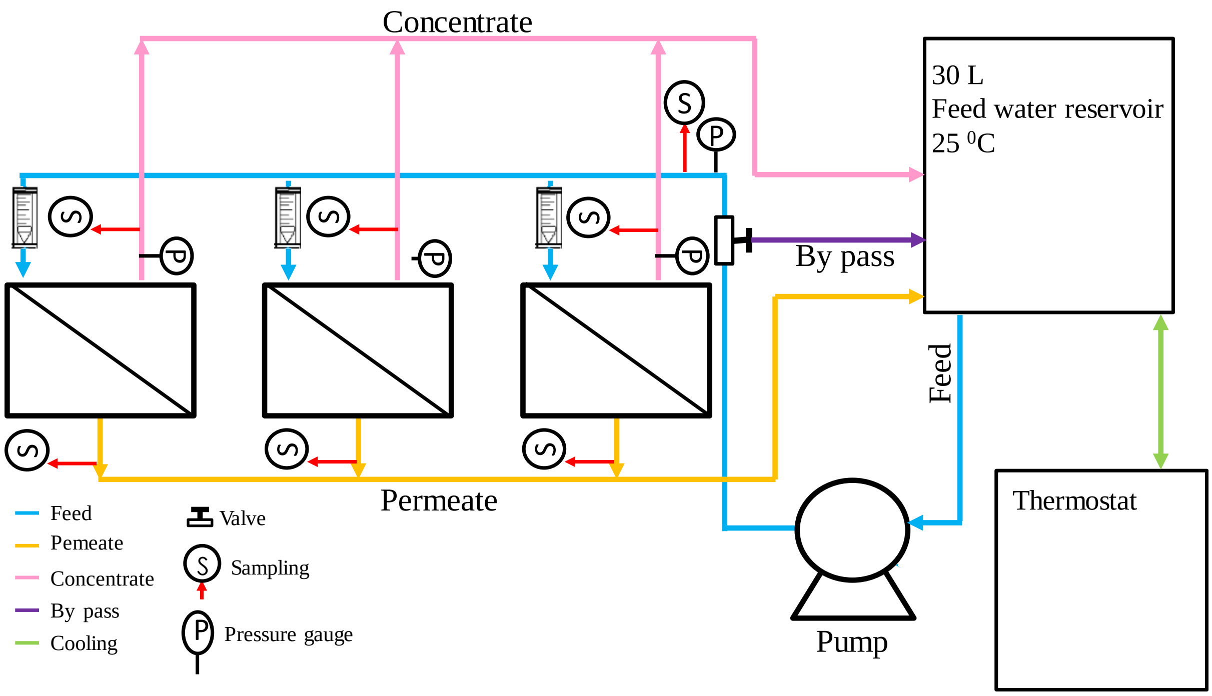

Figure S1 The schematic diagram of the cross-flow filtration system.

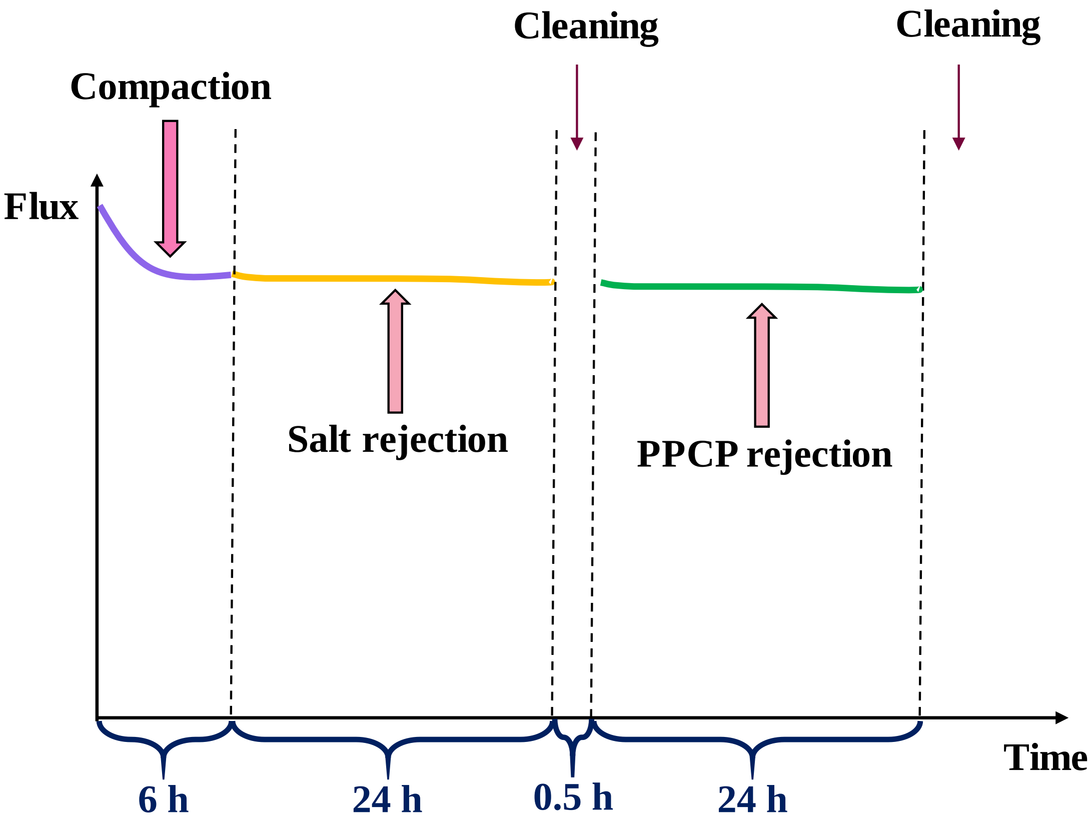

Figure S2 The schematic variation of permeate flux with filtration time.

References

[1] C.R. Wilke, P. Chang, Correlation of diffusion coefficients in dilute solutions, AIChE Journal, 1 (1955) 264-270.
